# Toward learning robust contrastive embeddings for binaural sound source localization

**DOI:** 10.3389/fninf.2022.942978

**Published:** 2022-11-16

**Authors:** Duowei Tang, Maja Taseska, Toon van Waterschoot

**Affiliations:** Department of Electrical Engineering (ESAT-STADIUS), KU Leuven, Leuven, Belgium

**Keywords:** manifold learning, non-linear dimension reduction, *siamese* neural network, binaural sound source localization, deep learning

## Abstract

Recent deep neural network based methods provide accurate binaural source localization performance. These data-driven models map measured binaural cues directly to source locations hence their performance highly depend on the training data distribution. In this paper, we propose a parametric embedding that maps the binaural cues to a low-dimensional space where localization can be done with a nearest-neighbor regression. We implement the embedding using a neural network, optimized to map points that are close to each other in the latent space (the space of source azimuths or elevations) to nearby points in the embedding space, thus the Euclidean distances between the embeddings reflect their source proximities, and the structure of the embeddings forms a manifold, which provides interpretability to the embeddings. We show that the proposed embedding generalizes well in various acoustic conditions (with reverberation) different from those encountered during training, and provides better performance than unsupervised embeddings previously used for binaural localization. In addition, the proposed method performs better than or equally well as a feed-forward neural network based model that directly estimates the source locations from the binaural cues, and it has better results than the feed-forward model when a small amount of training data is used. Moreover, we also compare the proposed embedding using both supervised and weakly supervised learning, and show that in both conditions, the resulting embeddings perform similarly well, but the weakly supervised embedding allows to estimate source azimuth and elevation simultaneously.

## 1. Introduction

Sound source localization is aiming to estimate a sound source position in terms of azimuth, elevation, and distance. A large part of the source localization literature focuses on the azimuth and elevation estimation only, hence this is also the scope we adopt in this paper. The human auditory system is capable of localizing acoustic signals using binaural cues such as the Interaural Phase Differences (IPDs) and Interaural Level Differences (ILDs) (Blauert, 1997). Computational localization algorithms in robot audition (Argentieri et al., 2015), hearing aid (Farmani et al., 2018), virtual reality (Keyrouz and Diepold, 2007), etc., aim at mimicking this process and therefore estimate the binaural cues from binaural microphone signals. The binaural microphones are typically two identical microphones that are mounted at the entries of two ear canals of an artificial head. In a sound source localization scenario, the human/artificial head together with the pinna and the torso act as filters that modify the incident sound waves. This filter effect is crucial for sound source localization, especially vertical sound source localization (i.e., elevation estimation), and can be characterized by the Head-related Transfer Function (HRTF) (Risoud et al., 2018).

Acoustic artifacts such as noise and reverberation, introduce uncertainties in the binaural cues. Although the existence of reverberation can aid distance localization (Risoud et al., 2018), the resulting noisy and reverberant binaural cues make sound source localization challenging. Traditionally, robustness to reverberation has been tackled with statistical model-based approaches (Mandel et al., 2010; May et al., 2011; Woodruff and Wang, 2012), which outperform lookup tables and template matching methods that rely on an anechoic assumption (Raspaud et al., 2010; Karthik and Ghosh, 2018). Some works propose to estimate the direct-path relative transfer function, which encodes the source azimuth information, in order to avoid the contamination of audio from reverberation noise, however, this type of methods highly rely on the onset of the source acoustic events (Li et al., 2016).

In contrast, data-driven approaches are able to learn the non-linear functions that map binaural cues to source locations (Datum et al., 1996). Recently, Deep Neural Networks (DNNs) has been used to learn the relationship between azimuth and binaural cues, by exploiting head movements to resolve the front-back ambiguity (Ma et al., 2017), and by combining spectral source models to robustly localize the target source in a multiple sources scenario (Ma et al., 2018). Additionally, a few works use DNNs to enhance the binaural features so that they can eliminate reverberation and additive noise (Pak and Shin, 2019; Yang et al., 2021). In Yalta et al. (2017) and Vecchiotti et al. (2019), the authors utilize DNNs to directly map the audio spectrogram or its raw waveform to the source azimuth in an end-to-end manner, which is also applicable to reverberant and noisy environments. However, those works only consider source azimuth estimation and the localization is done by classification (i.e., the predictions can only be in a pre-defined grid).

A different data-driven approach was used in Deleforge and Horaud (2012) and Deleforge et al. (2015), where the relationship between source locations and binaural cues was modeled with a probabilistic piecewise linear function. By learning the function parameters, sources can be localized by probabilistic inversion. An implicit assumption of the piecewise linear model in Deleforge and Horaud (2012) and Deleforge et al. (2015) is that similar source locations result in similar binaural cues. The same assumption is also used in non-parametric source localization algorithms based on manifold learning in Laufer et al. (2013) and Laufer-Goldshtein et al. (2015). In this paper, we focus on data-driven source localization approaches, inspired by low-dimensional manifold learning (Laufer et al., 2013; Laufer-Goldshtein et al., 2015).

Manifold learning in sound source localization is aiming to find a non-linear transformation that transforms acoustic measurements to a low-dimensional representation that preserves the source locality information. Manifold learning methods in Laufer et al. (2013) and Laufer-Goldshtein et al. (2015) rely on smoothness in the measurement space with respect to the underlying source locations, an assumption that might generalize poorly to varying acoustic conditions. The uncertainties in the binaural cue measurements introduced by reverberation, introduce variations in the measurement space neighborhoods that might not be consistent with their source locations. To preserve neighborhoods in term of the source location, we are inspired by the “*siamese”* neural network in the machine learning community that is optimized with a contrastive loss function (Hadsell et al., 2006). This particular model learns a similarity metric defined in the latent space (i.e., written digit classes and orientation of air plane pictures in Hadsell et al., 2006). This paradigm, which doesn't rely on an explicit neighborhoods definition in the measurement space, is suitable for problems that have a large amount of classes and in each class there are only a few training examples, such as face verification (Chopra et al., 2005; Taigman et al., 2014) and signature verification (Bromley et al., 1993), and can also be used in sound source localization. We have proposed and published earlier a regression method for binaural sound source localization based on the “*siamese”* neural network and contrastive loss in Tang et al. (2019). This method converts binaural cues into a low-dimensional embedding, and there is a small Euclidean distance between the embeddings obtained from binaural cues of similar source locations. A similar work using triplet loss somewhat resembles our idea (Opochinsky et al., 2019), but in their work, a model directly maps the binaural cues to source location predictions, and pre-defined proximity for both positive and negative cases (i.e., points with similar and dissimilar source locations) have to be present at the same time for the triplet loss.

In this paper, we first propose an update on the model architecture introduced in Tang et al. (2019), and then validate its robustness with respect to three aspects:

1. mismatched audio content between the training and testing sets,
2. the presence of unknown reverberation and noise,
3. and the availability of only a small amount of annotated training data,

through abundant experiments in fixed and varying acoustic scenarios, respectively. Afterwards, we extend our method to a weakly supervised learning scheme, where the annotation of source directions (i.e., azimuth and elevation) is no longer required for training the embeddings, but only the relative source position proximity is needed for any pair of training examples. Unlike the supervised approach proposed in Tang et al. (2019), which treats azimuth and elevation estimation in two separate tasks, this weakly supervised embedding can be used to estimate both the source azimuth and elevation at the same time, and providing a good visualization of the manifold.

The proposed methods have potential in a number of practical applications where the location of a sound source is to be identified, for example in signal processing front-ends for hearing aids, and in an intelligent interactive dialogue systems, to localize the speaker for denoizing beamformers, or for a synthesizer to render stereo sounds. Note that the proposed methods start from binaural signal features, which implies that binaural rather than bilateral hearing aids are required when using these methods for sound source localization in hearing aid systems, and the issue of binaural hearing aids that need to transmit and synchronize the binaural features needed for this model is beyond the scope of this paper. Yet there is a large body of research literature that addresses this issue, and the reader is referred to Kreisman et al. (2010), Ibrahim et al. (2013), Wei et al. (2014), and Geetha et al. (2017).

The paper is organized as follows. In Section 2, we first revise the binaural cue extraction and formulate the source localization problem. Then, in Section 3, we provide a brief overview of the related manifold learning work that has been applied in binaural sound source localization. Next, the proposed method is presented in Section 4 and finally, experimental results are shown in Section 5.

## 2. Data model and problem formulation

### 2.1. Binaural cue extraction

Let *s*_1_(τ) and *s*_2_(τ) denote the signals captured at the left and right microphones in a binaural recording setup in a noisy and reverberant environment. In this work, we extract the binaural cues in the Short-time Fourier transform (STFT) domain, as in Raspaud et al. (2010) and Deleforge et al. (2015).

Let *S*_1_(*t, k*) and *S*_2_(*t, k*) denote the STFT coefficients of *s*_1_(τ) and *s*_2_(τ), where *t* and *k* are the time frame and frequency index, respectively. At a time-frequency bin (*t, k*) an ILD α_*tk*_ and an IPD ϕ_*tk*_ are defined as


(1) $$\begin{array}{l} \alpha_{tk}=20\;{\text{log}}_{10}\frac{\left|S_{1}\left(t,k\right)\right|}{\left|S_{2}\left(t,k\right)\right|},\;\phi_{tk}=∠\frac{S_{1}\left(t,k\right)}{S_{2}\left(t,k\right)}. \end{array}$$

Assuming that a single sound source is active, we follow the binaural feature extraction approach from Deleforge et al. (2015), and compute time-averaged ILDs and IPDs across *T* frames as follows


(2) $$\begin{array}{l} a_{k}=T^{-1}\overset{T}{\underset{t=1}{\sum }}\alpha_{tk},\;p_{k}=T^{-1}\overset{T}{\underset{t=1}{\sum }}\text{exp}\left(j\phi_{tk}\right). \end{array}$$

By concatenating the ILDs, and the real and imaginary parts of the IPDs in selected frequency ranges [*k*_1_, *k*_2_] and [*k*_3_, *k*_4_], the binaural information is summarized in a measurement vector $\text{x}\in X\subset {\mathbb{R}}^{D}$,


(3) $$\begin{array}{l} \text{x}={\left[a_{k_{1}},\ldots ,a_{k_{2}},\;R\left\{p_{k_{3}}\right\},I\left\{p_{k_{3}}\right\},\ldots ,R\left\{p_{k_{4}}\right\},I\left\{p_{k_{4}}\right\}\right]}^{\text{T}} \end{array}$$

with dimensionality *D* = *k*_2_−*k*_1_+2(*k*_4_−*k*_3_).

It is known that IPDs carry reliable location cues below 2 kHz (Blauert, 1997), while ILDs contribute to localization at higher frequencies as well (Deleforge et al., 2015). Hence, we used the ranges $\frac{f_{s}}{K}\left[\tilde{k_{1}},\tilde{k_{2}}\right]=\left[200;7,000\right]$ Hz for ILDs and $\frac{f_{s}}{K}\left[\tilde{k_{3}},\tilde{k_{4}}\right]=\left[200;2,500\right]$ Hz for IPDs, where *f*_*s*_ denotes the sampling frequency and *K* is the Discrete Fourier transform (DFT) size used in the STFT, and $k_{i}=\text{round}\left({\tilde{k}}_{i}\right),i=1,2,3,4$, where the round() operation rounds ${\tilde{k}}_{i}$ to the closest integer. For a typical audio recording with sampling rate *f*_*s*_ = 16 kHz, and the DFT size *K* = 1, 024, the dimensionality *D* is equal to 729 (i.e., a 729-dimensional feature vector ***x***).

### 2.2. Measurement to embedding transformation

From the above binaural cue extraction process, a pair of signals *s*_1_(τ) and *s*_2_(τ) is associated to a vector x∈X. We refer to X as the *measurement* space. Let the unknown source location be denoted by u∈U. We refer to U as the *latent space*. U is one-dimensional if one considers azimuth or elevation separately, or two-dimensional if the localization angles are considered simultaneously. Given a training set of *N* pairs $T={\left\{\left({\text{x}}_{i},{\text{u}}_{i}\right)\right\}}_{i=1}^{N}$, the localization problem consists of finding a function *h*


(4) $$\begin{array}{l} û=h\left(\text{x}\right),\;h:X\to U. \end{array}$$

that accurately maps measurements to latent variables. Although, one can implement *h* with a powerful non-linear model (e.g., a DNN), the proposed approach of first transforming the measurement space to an embedding space and then performing the localization in the embedding space comes with several advantages:

1. Learning the transformation from measurement space to embedding space does not necessarily require the latent space annotation information, thus enables the possibility of semi-supervised learning and weakly supervised learning.
2. The low-dimensional embedding can preserve the latent space neighborhood relationships (in which the Euclidean distance in the embedding space roughly corresponds to the latent space “semantic” relationship) and the embedding eliminates useless information, which can be used to study or visualize the latent space structure. A vanilla example of this is the Principal Component Analysis (PCA).
3. By learning the structure of the latent space, the training of the model will be less dependent on the distribution of the training data. In contrast, if the mapping from measurement space to latent space is learned directly, the model is more likely to over-fit to the dense part of the training data and its generalization capability decreases when there is not enough annotated training data.

Therefore, our main objective in this work is to learn an embedding function *f* that maps the vectors ***x*** to a low-dimensional space which preserves latent space neighborhoods, i.e.,


(5) $$\begin{array}{l} \text{z}=f\left(\text{x}\right),\;f:X\to Z\subset {\mathbb{R}}^{d},\;d<<D. \end{array}$$

We propose a neural network framework to learn a parametric function *f* that satisfies these properties both in a supervised and weakly supervised manner. A nearest-neighbor regression function h:Z→U is then used for localization.

## 3. Baseline manifold learning method

If the microphone location in a given room is fixed, the authors in Laufer-Goldshtein et al. (2015) showed that features extracted from binaural signals can be embedded in a low-dimensional space Z, in a way that recovers source locations. The framework in Laufer-Goldshtein et al. (2015) is based on unsupervised manifold learning, in particular, *Laplacian eigenmaps* (LEM) (Belkin and Niyogi, 2003).

The Laplacian Eigenmaps (LEM) method defines the neighborhood relationships of the data using a similarity matrix ***K*** ∈ ℝ^*N*×*N*^, with entries ***K***[*i, j*] related to the Euclidean distances ||***x***_*i*_−***x***_*j*_||_2_ between feature vectors *x*_*i*_ and *x*_*j*_, with *i, j*∈[1, *N*]. One way to compute ***K*** is using nearest-neighbors, i.e., ***K***[*i, j*] = ***K***[*j, i*] = 1 if ***x***_*i*_ is among the *M* nearest neighbors of ***x***_*j*_, or if ***x***_*j*_ is among the *M* nearest neighbors of ***x***_*i*_ (in Euclidean distance). A second way is using an exponentially decaying kernel function, such as the Gaussian kernel


(6) $$\begin{array}{l} \text{K}\left[i,j\right]=\text{exp}\left(-\frac{||x_{i}-x_{j}|{|}_{2}^{2}}{\varepsilon }\right), \end{array}$$

where ε is the kernel bandwidth. Such kernel is used for source localization in Laufer-Goldshtein et al. (2015).

Given the similarity matrix ***K***, the neighborhood-preserving optimization problem of LEM to find the embeddings *z*_1_, *z*_2_, …, *z*_*N*_ is given by (Belkin and Niyogi, 2003)


(7) $$\begin{array}{l} \begin{array}{cc} \underset{{\text{z}}_{1},\ldots ,{\text{z}}_{N}}{\arg\min}\; & \overset{N}{\underset{i,j=1}{\sum }}||{\text{z}}_{i}-{\text{z}}_{j}|{|}_{2}^{2}\text{K}\left[i,j\right],\\ \text{subject to } & {\text{Z}}^{T}\text{D}\text{Z}=\text{I} \end{array} \end{array}$$

which enforces that points *x*_*i*_, *x*_*j*_ with large similarity ***K***[*i, j*], are to be mapped to points *z*_*i*_, *z*_*j*_ with a small Euclidean distance ||***z***_*i*_−***z***_*j*_||_2_ where ***D*** is a diagonal matrix with entries $\text{D}\left[i,i\right]=\overset{N}{\underset{j=1}{\sum }}\text{K}\left[i,j\right]$.

The optimization problem (7) has a closed-form solution, given by the eigenvectors of ***P*** = ***D***^−1^***K*** corresponding to the largest eigenvectors. If ${\left\{{\text{ψ}}_{i}\right\}}_{i=1}^{N}$ denote the eigenvectors of ***P***, with eigenvalues 1 = λ_1_> λ_2_ ≥…, ≥ λ_*N*_, the *d*-dimensional LEM embedding is given by (Belkin and Niyogi, 2003)


(8) $$\begin{array}{l} {\text{z}}_{i}=f\left({\text{x}}_{i}\right)={{\text{ψ}}_{2}\left[i\right],{\text{ψ}}_{3}\left[i\right],\ldots ,{\text{ψ}}_{d+1}\left[i\right]}^{\text{T}}, \end{array}$$

where the constant eigenvector **ψ**_1_ is not included (Chung, 1997; Belkin and Niyogi, 2003) and [*i*] denotes the vector element index. The LEM embedding *f* is non-parametric, and the low-dimensional representation ***z*** of a new measurement ***x*** is obtained as a linear combination of the training points ${\left\{{\text{z}}_{i}\right\}}_{i=1}^{N}$ (Bengio et al., 2003). However, this procedure is often insufficiently accurate and represents a disadvantage of LEM and of spectral embeddings in general. One can include every new testing data and re-run the unsupervised training to get a more accurate representation for the new testing data, however, this may prolong the training time, especially for large datasets, and due to the fact that the kernel matrix ***K*** is *N*×*N*, the computation of eigenvectors will dramatically increase for a large *N*.

Besides the promising performance of spectral embeddings for localization (Laufer et al., 2013; Laufer-Goldshtein et al., 2015; Taseska and van Waterschoot, 2019), their major drawback is the assumption that the neighborhoods in the measurement space are consistent with the source locations. Although the assumption is shown to hold when all signals are recorded in one room for fixed microphone locations (Deleforge and Horaud, 2012; Laufer-Goldshtein et al., 2015; Taseska and van Waterschoot, 2019), this is not the case when the signals are filtered by various acoustic channels in different enclosures.

## 4. Contrastive embedding for localization

We propose a parametric embedding, designed to preserve neighborhoods in terms of sound source locations. Such embeddings are robust to unseen room reverberation and small training set size (e.g., when the training set does not contain the complete latent space annotations). The proposed framework firstly includes the definition of the neighborhoods, which can be supervised (Section 4.1) or weakly supervised (Section 4.2) depending on whether one uses the azimuth/elevation label or the source relative proximity. Secondly it includes the transformation from the measurement space to the embedding space by training a DNN which is optimized on a contrastive loss function (Sections 4.3 and 4.4). Finally the sound source localization will be performed in the embedding space using nearest-neighbor regression (Section 4.5).

### 4.1. Supervised neighborhoods definition

Consider two labeled measurements (***x***_*i*_, *u*_*i*_) and (***x***_*j*_, *u*_*j*_) where *u*_*i*_ and *u*_*j*_ are denoted as scalars since we estimate azimuth and elevation separately. To avoid the phase wrapping ambiguity, we define $d_{u}\left(u_{i},u_{j}\right)=\min\left(\left|u_{i}-u_{j}|,360^{•}-|u_{i}-u_{j}\right|\right)$ denote the shortest possible distance in the latent space U, where *u*_*i*_, *u*_*j*_ corresponds to the source azimuth or elevation angles in degree. A neighborhood indicator *y*_*ij*_∈{0, 1} is defined as


(9) $$\begin{array}{l} y_{ij}=\{\begin{array}{c} 0,\text{if}d_{u}\left(u_{i},u_{j}\right)>\epsilon_{u}\\ 1,\text{if}d_{u}\left(u_{i},u_{j}\right)\leq \epsilon_{u}, \end{array} \end{array}$$

for a user-defined threshold angle ϵ_*u*_.

### 4.2. Weakly supervised neighborhoods definition

As an alternative to directly using the latent space label information to define the neighborhoods, we can also use the relative proximity between sound sources. Here, we only consider the sound sources at the ball with radius Φ and centered at the receiver, or sources whose relative position to the receiver can be found (then the source locations can be firstly projected onto a ball with radius Φ around the receiver by distance normalization).

In order to define the weakly supervised neighborhoods, we can use the physical distance *d*_*s*_(*S*_*i*_, *S*_*j*_) between two sound sources *S*_*i*_ and *S*_*j*_ which corresponds to the Euclidean distance between the Cartesian coordinate vectors of *S*_*i*_ and *S*_*j*_. Similarly,


(10) $$\begin{array}{l} y_{ij}^{\prime }=\{\begin{array}{c} 0,\text{if}d_{s}\left(S_{i},S_{j}\right)>\epsilon_{s}\\ 1,\text{if}d_{s}\left(S_{i},S_{j}\right)\leq \epsilon_{s}, \end{array} \end{array}$$

for a user-defined threshold distance *ϵ*_*s*_. The threshold *ϵ*_*s*_ and *ϵ*_*u*_ are related as *ϵ*_*s*_ represents the arc length of the angle *ϵ*_*u*_ on a circle with radius Φ and hence,


(11) $$\begin{array}{l} \epsilon_{s}\approx \epsilon_{u}\cdot \text{Φ}\cdot \pi /180^{•} \end{array}$$

In particular, in our proposed method, one can also implicitly define the similarity indicator $y_{ij}^{\prime }$ by using it as a training data label. For example, consider a scenario when multiple recordings are acquired from excitations at each of the pre-defined sound source locations, then $y_{ij}^{\prime }$ equals to 1 for recordings acquired at the same or at close source locations, and $y_{ij}^{\prime }$ equals to 0 for recordings acquired at different or far source locations.

### 4.3. Contrastive loss

We seek to learn a parametric function $f_{W}:X\to Z\subset {\mathbb{R}}^{d}$, with parameters *W*, that maps ***x***_*i*_ and ***x***_*j*_ to their low-dimensional embeddings ***z***_*i*_ and ***z***_*j*_. If *y*_*ij*_ = 1, the Euclidean distance ||***z***_*i*_−***z***_*j*_||_2_ should be small, and if *y*_*ij*_ = 0, then ||***z***_*i*_−***z***_*j*_||_2_ should be large. For a given embedding function *f*_*W*_, we have


(12) $$\begin{array}{l} ||{\text{z}}_{i}-{\text{z}}_{j}|{|}_{2}=||f_{W}\left({\text{x}}_{i}\right)-f_{W}\left({\text{x}}_{j}\right)|{|}_{2}. \end{array}$$

A *contrastive loss function* over the parameters *W*, tailored for neighborhood preservation has been proposed in Hadsell et al. (2006) for non-linear dimensionality reduction, and is given by


(13) $$\begin{array}{ll} L(W)=\overset{N}{\underset{i=1}{\sum }}\overset{N}{\underset{j=1}{\sum }}(y_{ij}||f_{W}(x_{i})-f_{W}(x_{j})|{|}_{2}^{2}\\ \;+(1-y_{ij})\text{max}{\left(0,\mu_{ij}-||f_{W}(x_{i})-f_{W}(x_{j})|{|}_{2}\right)}^{2}). & \;\left(13\right) \end{array}$$

The parameter μ_*ij*_ is a positive real-valued margin, such that ^μ_*ij*_^/_2_ can be interpreted as the same radius of circles centered on ***z***_*i*_ and ***z***_*j*_. If the circles intersect and *y*_*ij*_ = 0, the two dissimilar pairs are too close in the embedding space, thus increasing the *contrastive loss* in (14). On the other hand, if *y*_*ij*_ = 1, large distances are penalized, enforcing *f*_*W*_ to preserve neighborhoods.

Intuitively speaking, during the training, each example in a mini-batch is subjected to two “forces.” One force is between the similar pairs, pulls them closer to each other in the embedding space. The other force between dissimilar pairs is repulsive and it pushes the dissimilar pair away from each other in the embedding space (if they are too close when ||*f*_*W*_(***x***_*i*_)−*f*_*W*_(***x***_*j*_)||_2_ < μ_*ij*_). During training, the embeddings are moving according to the forces they encounter, and thus will eventually lead to an equilibrium (i.e., convergence). Globally, the embedding space convergences to a manifold. Since the forces are subjected to latent space similarities, this will result in meaningful distances between each pair of embeddings (i.e., the distance between a pair of embeddings somewhat indicates the proximity of their corresponding sound sources).

It is important to note that in Hadsell et al. (2006), where the *contrastive loss* was first proposed for classification, μ_*ij*_≡μ is a constant margin. In our application, the latent space of azimuths and elevations is continuous. To accurately preserve its geometry, we propose an adaptive margin as follows,


(14) $$\begin{array}{l} \mu_{ij}=\frac{\text{exp}\left(d_{ij}\right)}{\text{exp}\left(d_{ij}\right)+1}. \end{array}$$

As *d*_*ij*_ decreases, the margin μ_*ij*_ decreases as well. One can compute *d*_*ij*_ either in a supervised manner using the azimuth/elevation, thus *d*_*ij*_ = *d*_*u*_(*u*_*i*_, *u*_*j*_), or in a weakly supervised manner, where *d*_*ij*_ = *d*_*s*_(*S*_*i*_, *S*_*j*_). In the case that there is no quantitative measure in the latent space, a constant margin can be used (e.g., μ = 1).

### 4.4. Learning the embedding

**Figure 1:**
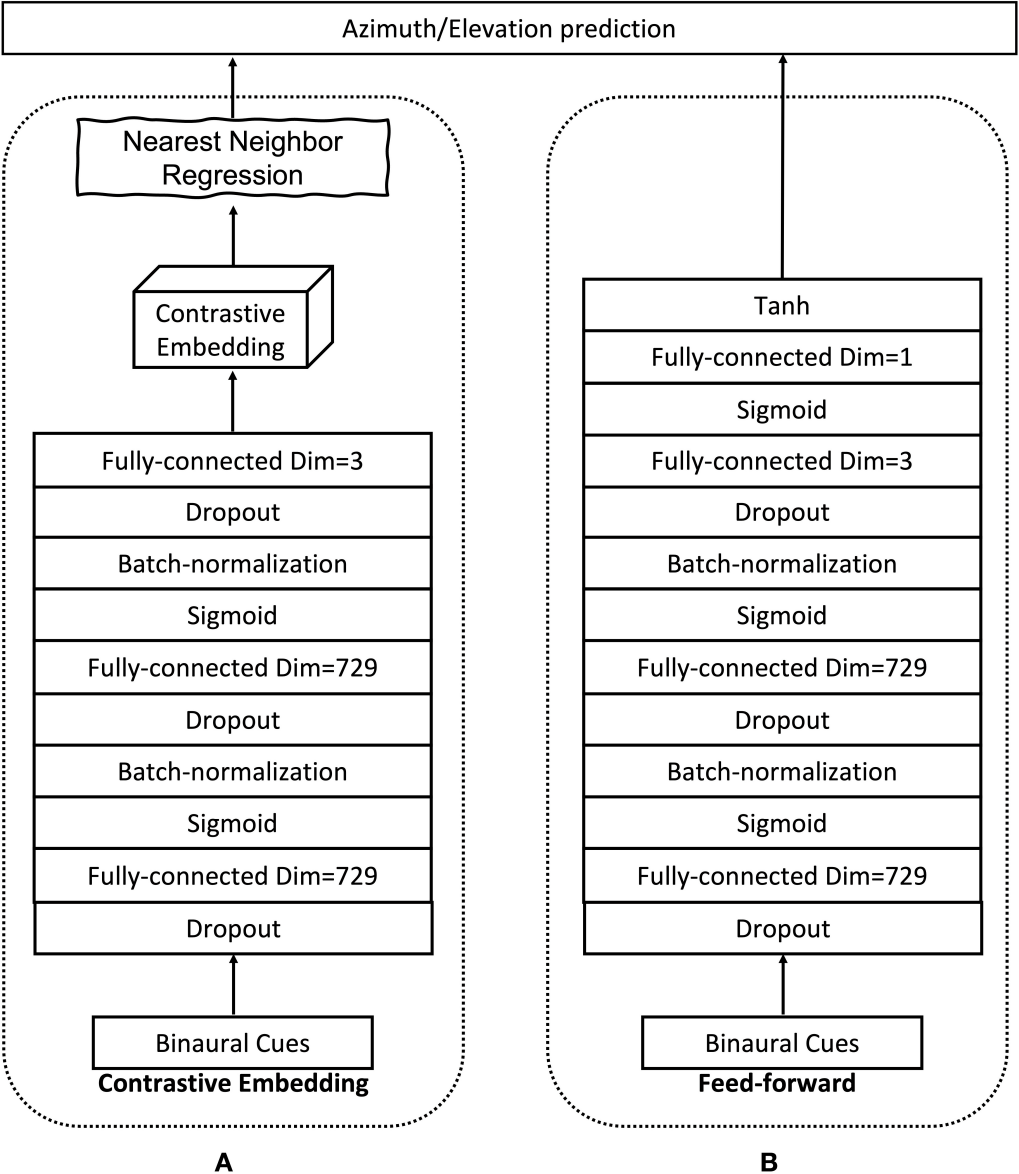
Model architecture. The proposed contrastive embedding model in **(A)**, and the feed-forward model in **(B)**.

We implement *f*_*W*_ with a DNN as shown in Figure 1A. The DNN architecture consists of two fully-connected hidden layers with *D* neurons in each layer. Between the fully connected layers, we add batch-normalization layers (Ioffe and Szegedy, 2015) to speed up the convergence and dropout layers to prevent the model from over-fitting (Srivastava et al., 2014). The output layer has three neurons, corresponding to a three-dimensional embedding space, i.e., *d* = 3. The hidden neurons have *Sigmoid* non-linear activations, and the output neurons have linear activations. In order to train the DNN model to minimize the cost function in (14), we use the *siamese* architecture that was proposed in Bromley et al. (1993) and used for various tasks in Chopra et al. (2005) and Hadsell et al. (2006). This special DNN architecture consists of two identical branches that are sharing the same model parameters. Taking a pair (***x***_*i*_, ***x***_*j*_) as an input, the measurements ***x***_*i*_ and ***x***_*j*_ are passed through the branches (one per branch) and hence produce their corresponding embeddings ***z***_*i*_ and ***z***_*j*_. Then the cost is evaluated in (14) using the neighborhood indicator *y*_*ij*_ and the outputs ***z***_*i*_ and ***z***_*j*_ of the branches. Finally, the gradient per model parameter is calculated and back-propagated to update the model parameters. Depending on which definition for the neighborhood indicator is used, we call the corresponding embedding Supervised Contrastive Embedding (SCE) if the supervised neighborhoods definition is used, or Weakly-supervised Contrastive Embedding (WSCE) if the weakly supervised neighborhoods definition is used.

A key aspect of the proposed framework is the selection of pairs (***x***_*i*_, ***x***_*j*_) for training. For small datasets, one could consider all pairs and proceed with training on all training data pairs. However the polynomial growth of the number of pairs results in memory problems even for moderately large datasets. To solve this problem, we use mini-batches and calculate the neighborhood indicator *y*_*ij*_ for every pair of examples in each mini-batch. To be noted, we suggest to choose a large enough batch size so that there are both similar pairs and dissimilar pairs in one batch. Because a randomly selected mini-batch generally contains examples from sources of different locations (i.e., those examples will be defined as dissimilar pairs), if the batch size is too small, the probability of having similar pairs in a batch will be very low, so that the loss will be inaccurately evaluated and thus slow down the convergence rate. Intuitively, if there is no similar pair in a batch, the embeddings will not be subjected to a pulling force to their similar points. This would lead to the embeddings that just randomly reside in the embedding space and form local clusters.

### 4.5. Nearest-neighbor localization

Once the weights of *f*_*W*_ are optimized, we compute the embedding of a new ***x*** by a forward-pass through the DNN model. Let ***z***_1_, …, ***z***_*K*_ denote the *K* nearest-neighbors of ***z*** in the training set. The latent variable (azimuth or elevation) is then estimated as


(15) $$\begin{array}{l} û=\overset{K}{\underset{i=1}{\sum }}w_{i}u_{i},\text{with}w_{i}=\frac{\text{exp}\left(-\frac{||\text{z}-{\text{z}}_{i}|{|}_{2}^{2}}{\varepsilon }\right)}{\overset{K}{\underset{j=1}{\sum }}\text{exp}\left(-\frac{||\text{z}-{\text{z}}_{j}|{|}_{2}^{2}}{\varepsilon }\right)}. \end{array}$$

The bandwidth ε of the exponential kernel is obtained as the median of the squared distances from the *K* neighbors, i.e.,


(16) $$\begin{array}{l} \varepsilon =\text{median}\left(||\text{z}-{\text{z}}_{1}|{|}_{2}^{2},\ldots ,||\text{z}-{\text{z}}_{K}|{|}_{2}^{2}\right). \end{array}$$

Note that if the embedding is accurately preserving neighborhoods, the choice of regression weights is not critical. For instance *w*_*i*_ can be inversely proportional to $||\text{z}-{\text{z}}_{i}|{|}_{2}^{2}$. However, in our experiments, the latter generally leads to less accurate location estimates than exponentially decaying weights.

## 5. Experiments

### 5.1. Experimental settings

To evaluate the proposed SCE in terms of the localization error and robustness, we compare the SCE with two baseline methods:

1. The LEM embeddings (Laufer et al., 2013; Laufer-Goldshtein et al., 2015) with nearest neighbor localization.
2. A feed-forward neural network which is optimized with the Mean Squared Error (MSE) loss. This feed-forward neural network has the same structure as one of the branches in the proposed *siamese* structure except for an additional output layer with *tanh* activation functions that outputs the source location predictions, shown in Figure 1B. Since the *tanh* activation function has a range of (−1, 1), we normalize the training labels also to the same range by $\hat{u_{i}}=u_{i}/180^{•}$, and *i* = 1…*N*. Note that, the original labels have a range [−180, 180°]. During testing, the feed-forward predictions are firstly converted back to degree before calculating the localization errors.

As the neighborhoods for LEM are defined in the input space, a single embedding is used to estimate both azimuth and elevation. Similarly, our proposed method can be trained to estimate azimuth and elevation simultaneously as well by using the weakly supervised neighborhoods definition introduced in Section 4.2. However, a system with two separately trained embeddings might provide better results for the same amount of data, which we will compare for SCE and WSCE in the later experiments.

For the nearest neighbor regression in (16), *K* = 5 neighbors are used in all localization experiments. A few threshold values ϵ_*u*_ in (9) and (11) are tested for both azimuth and elevation. We choose ϵ_*u*_ in {5, 15, 30°} to have a big span so that we can evaluate its impact on the localization results. Essentially, ϵ_*u*_ is a hyper-parameter that can be tuned with a validation set. We implemented the LEM using a nearest neighbor kernel ***K*** with *M* = 10 nearest neighbors, which in our experiments, provided better results than the Gaussian kernel used in Laufer-Goldshtein et al. (2015) and Taseska and van Waterschoot (2019).

For DNN training, we use the Adam optimizer (Kingma and Ba, 2015) with a learning rate equal to 10^−3^ that is automatically halved if the validation performance does not improve after 20 epochs. The mini-batch size is set to 128, and this will result 8,128 pairs of measurements per mini-batch for training. We select the model based on the best validation performance, and then the selected model is used to calculate the testing set predictions.

All audio files are sampled at 16 kHz. To extract the ILD and IPD features, we use the STFT with a cosine window of 1,024 samples at 16 kHz, 75% overlapping.

### 5.2. Datasets

#### 5.2.1. Fixed acoustic conditions

With the first dataset, we want to verify the effectiveness of our proposed methods for preserving the locality information of the audio source when the training and the testing set have different audio content (and different spectral distribution). We employ the CAMIL dataset which consists of binaural recordings and was gathered using a Sennheiser MKE 2002 dummy head in a real-life reverberant room (i.e., a room with a few furnitures and background noise; Deleforge et al., 2015). To generate recordings that have different azimuth and elevation angles, a loudspeaker (i.e., the source) is placed at a fixed position, 2.7 m from the dummy head (i.e., the receiver). The dummy head is mounted on a step-motor which generates 10,800 pan-tilt states. This results in source azimuth and elevation angle in the range [−180, 180°] and [−60, 60°], respectively (with 2 ° resolution). To only evaluate the methods in localizing frontal sources, we select the recordings that have source azimuth and elevation angle in the range [−90, 90°] and [−45, 45°], respectively. The CAMIL dataset consists of a training set made using white noise (1 s per recording), and a testing set made using 1–5 s speech samples from the TIMIT corpus (Garofolo et al., 1993). We further randomly divide the whole training set into a smaller training set (consisting of 70% samples from the original training set), and a validation set (consisting of the remaining 30% samples from the original training set). Finally, spatially uncorrelated white noise with a Signal to Noise Ratio (SNR) of 15 dB is added to the testing set.

#### 5.2.2. Varying acoustic conditions

With the second dataset, we want to verify the robustness of the proposed methods for varying acoustic conditions. We use the VAST dataset (Gaultier et al., 2017) of simulated binaural room impulse responses of a KEMAR dummy head (Gardner and Martin, 1995; Schimmel et al., 2009). The training set consists of 16 different rooms with reverberation time 0.1–0.4 s. For each room we select spherical grids of source positions with radii 1, 1.5, and 2 m, centered at nine predefined receiver positions (inside each room). Similarly to the fixed acoustic conditions in Section 5.2.1, we use 70% of randomly selected data as the training set, and the remaining 30% as the validation set. The receiver's height is fixed at 1.7 m. Then two testing sets are provided:

- *Testing-set-1*: The source and receiver are placed at random positions in the same 16 rooms as the training set.
- *Testing-set-2*: The source and receiver are placed in shoebox rooms of random width and length between 3 × 2 and 10 × 4 m, with absorption profiles randomly picked from those of the training rooms. Those rooms have reverberation time 0.1–0.4 s.

All the training set's and testing sets' Head-related impulse responss (HRTFs) are simulated using the image source method (Allen and Berkley, 1979) and provided by the VAST dataset (Gaultier et al., 2017).

As in Section 5.2.1, we have only selected recordings that have frontal angles. To focus on the influence of the varying room acoustics while exciting all frequencies, 2 s white noise source signals were considered in this experiment.

### 5.3. SCE for unidimensional source localization

#### 5.3.1. Tuning the dropout rate

**Figure 2:**
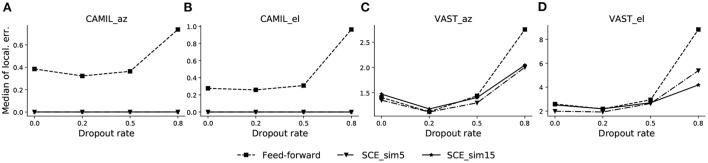
Validation performance across different dropout rates for CAMIL validation set **(A,B)**, and VAST validation set **(C,D)**. The dropout rate is tested for both the proposed SCE with similarity threshold angle ϵ u equal to 5 and 15 (denoted by_sim5 and _sim15, respectively), and the baseline feed-forward method. Both methods are tested for source azimuth (denoted by _az) and elevation (denoted by _el) estimation. Localization errors are in degrees.

We first determine an optimal dropout rate for both the SCE method and the feed-forward model by line search. We test dropout rate values in {0.0, 0.2, 0.5, 0.8}, and similarity threshold values ϵ_*u*_ for SCE equals to 5 and 15° (denoted by “_sim5” and “_sim15,” respectively). The azimuth/elevation localization error of the validation sets for both the CAMIL dataset and the VAST dataset are plotted in Figure 2.

In Figures 2A,B, the azimuth and elevation estimation results for the CAMIL dataset are illustrated, respectively. We can observe that the SCE has better validation performance than the feed-forward model for all testing dropout rates, and its localization error is essentially equal to zero when using either similarity threshold value, i.e., 5 or 15°. The feed-forward model exhibits a clear concave curve in median localization error and has the lowest localization error at the dropout rate value of 0.2, thus indicating that a dropout rate equal to 0.2 is an optimal value for the feed-forward method.

In Figures 2C,D, the median azimuth and elevation localization error for the VAST dataset are illustrated respectively. Both the SCE and the feed-forward model in this case exhibit a concave curve in median localization error and they both exhibit an optimal dropout rate of 0.2. We also observe that, in the VAST azimuth validation performance, the SCE_sim5 performs equally well as the feed-forward model when dropout rate is 0.2, which is slightly better than SCE_sim15. In the elevation estimation, SCE_sim5 performs the best over the feed-forward model and SCE_sim15.

Based on the validation results, we choose the dropout rate equal to 0.2 for both the SCE and the feed-forward methods for the next experiments.

#### 5.3.2. Comparison with the baseline

In this experiment, we compare the localization performance of the proposed SCE with the baseline LEM embedding and the feed-forward model. For the proposed SCE, we evaluate a small threshold angle (i.e., ϵ_*u*_ = 5 °) and a large threshold angle (i.e., ϵ_*u*_ = 15 °), denoted by “_sim5” and “_sim15,” respectively.

**Figure 3:**
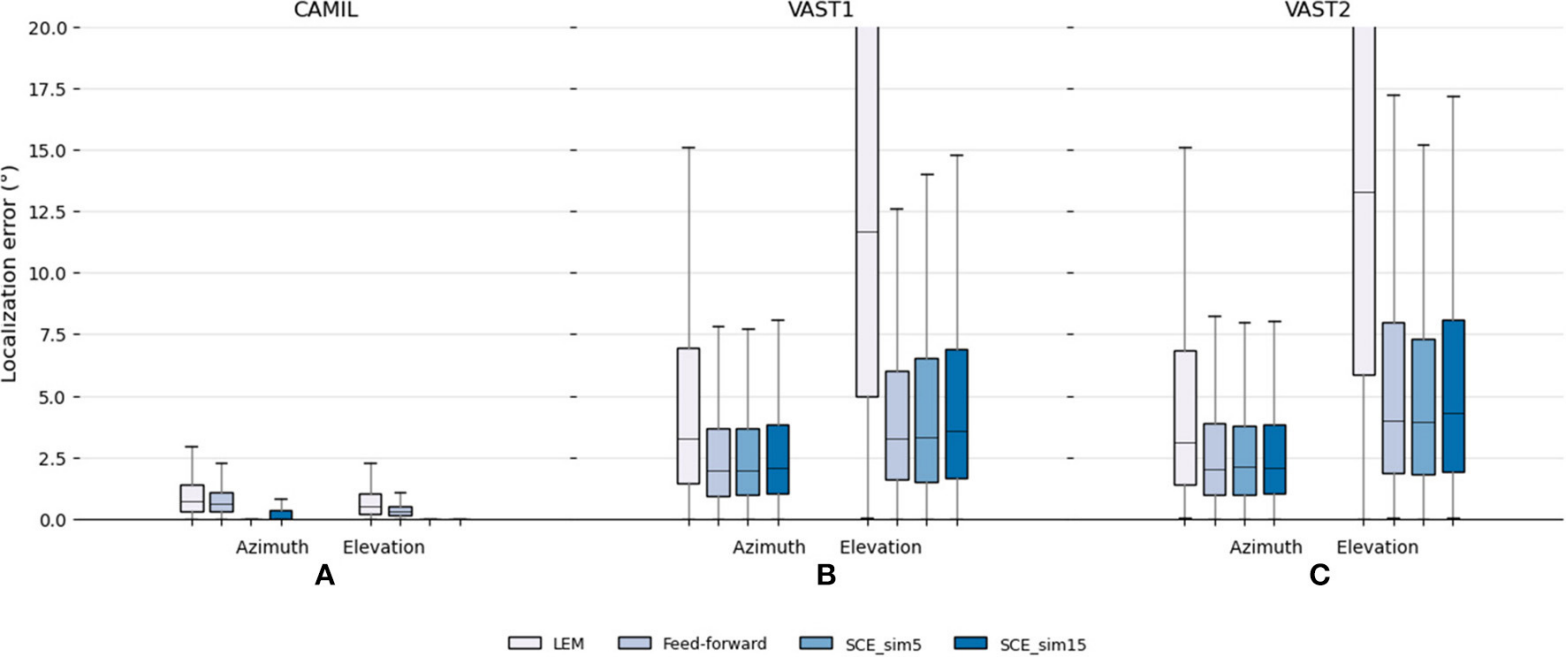
Testing set localization performance for the baseline LEM, the baseline feed-forward and the proposed SCE methods. Localization errors in **(A)** the fixed acoustic condition using the CAMIL testing set, and **(B,C)** the localization performance in the varying acoustic condition using the VAST testing sets. “_sim5” and “_sim15” denote the use of similarity threshold angles ϵ_*u*_ equal to 5 and 15° , respectively.

The testing set results are illustrated in Figure 3. It can be seen that in the fixed acoustic condition with the CAMIL dataset, the proposed SCE performs better than the LEM embedding and the feed-forward model in terms of median error and maximum error. Especially when using the small similarity threshold, the SCE performs excellent, as the SCE_sim5 has almost zero median error in azimuth and elevation estimations. It can also be noted that the feed-forward model performs slightly better than the LEM embedding, with a median error equal to 0.61 and 0.29° for azimuth and elevation respectively, whereas the LEM model has median errors equal to 0.72 and 0.49° for azimuth and elevation, respectively. In summary, in the fixed acoustic condition, the proposed SCE can almost perfectly preserve the source location information even when reverberation and additive white noise are present, while the feed-forward model performs better than the LEM embedding, but both exhibit some estimation error. This could be due to the fact that the feed-forward model highly depends on the training data, and due to the presence of audio content mismatch between the training and testing sets, the feed-forward model has some difficulty to generalize to unseen audio contents, thus negatively influencing the localization performance.

In the varying acoustic conditions with the VAST testing sets, the proposed SCE_sim5 performs slightly better than the SCE_sim15 and equally well as the feed-forward model. The SCE_sim5 and feed-forward model achieve VAST testing-set-1 azimuth median errors equal to 1.96 and 1.95°, VAST testing-set-1 elevation median errors equal to 3.32 and 3.24°, VAST testing-set-2 azimuth median errors equal to 2.1 and 2.01°, and VAST testing-set-2 elevation median errors equal to 3.94 and 3.99°, respectively. Since in the various acoustic conditions, the source excitations are white noise in both the training and testing set, the SCE and the feed-forward model can both generalize well to unseen acoustic environments, and show robustness toward reverberation and noise.

The LEM embedding performs the worst in the presence of various reverberations. It achieves median errors equal to 3.3 and 11.7 for azimuth and elevation in VAST test-set-1, respectively, and 3.1 and 13.3 for azimuth and elevation in VAST test-set-2, respectively. This may indicate that the LEM, which is easily affected by geometric distortion in the measurements, is not robust to reverberation.

#### 5.3.3. Reduced training-set

**Figure 4:**
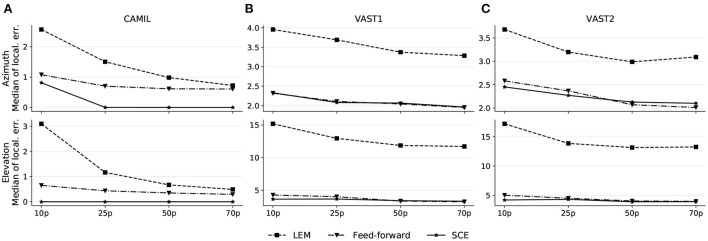
Localization performance on the CAMIL testing set **(A)**, the VAST testing-set-1 **(B)**, and the VAST testing-set-2 **(C)**. 10p, 25p, 50p, and 70p denote the cases when 10, 25, 50, and 70% of the original training data is used, respectively.

A common problem related to data-driven methods is the model generalizability, or in other words, how can a trained model generalize to unseen data. In the source localization scenario, the training set may not include training recordings from every pair of azimuth/elevation angles, hence it is desirable that the model can somehow interpolate the predictions that lie in-between the training points. In this experiment, we are aiming to evaluate the robustness of the proposed SCE toward the training size. With a smaller training size, there will be more source locations that are not included in the training. We use a similarity threshold angle ϵ_*u*_ = 5 ° for SCE in this experiment and all methods are conducted with 10, 25, 50, and 70% randomly selected training sets. The median localization errors are illustrated in Figure 4.

As illustrated by these results, all methods show a decreasing trend in localization error when a larger training set is used, however, the median localization error of the proposed SCE does not vary much with the changing size of the training set, and shows a flatter pattern. Although in the fixed acoustic condition, SCE results a in a higher median error when 10% of the training set is used (median azimuth error equal to 0.81°) than when a larger training set is used, the error is still lower than for the other two methods (as feed-forward and LEM achieve median azimuth errors equal to 1.08° and 2.56° respectively when 10% of the training data is used). This allows to conclude that the SCE is more robust to the use of training data that not cover the entire latent space.

The results allow us to hypothesize that the proposed SCE, leveraged by the contrastive loss and the adaptive margin (see Section 4.3), is aiming to learn a similarity metric between input binaural cues from the latent space. This similarity metric implies that the underlining structure in the latent space is robust to unseen source locations. In contrast, the feed-forward model tends to transform the measurement space to an abstract high-level space in which the Euclidean distance between embeddings is not necessarily a similarity metric, and thus it is difficult to infer the unseen source locations from this embedding space.

### 5.4. WSCE for multidimensional source localization

The LEM embedding as well as the proposed WSCE are capable of estimating the sound source azimuth and elevation simultaneously. It should be noted that both the proposed WSCE and the LEM need source annotations in order to localize new examples under the nearest-neighbor localization framework, thus the localization phase is still a supervised learning task for both methods.

**Figure 5:**
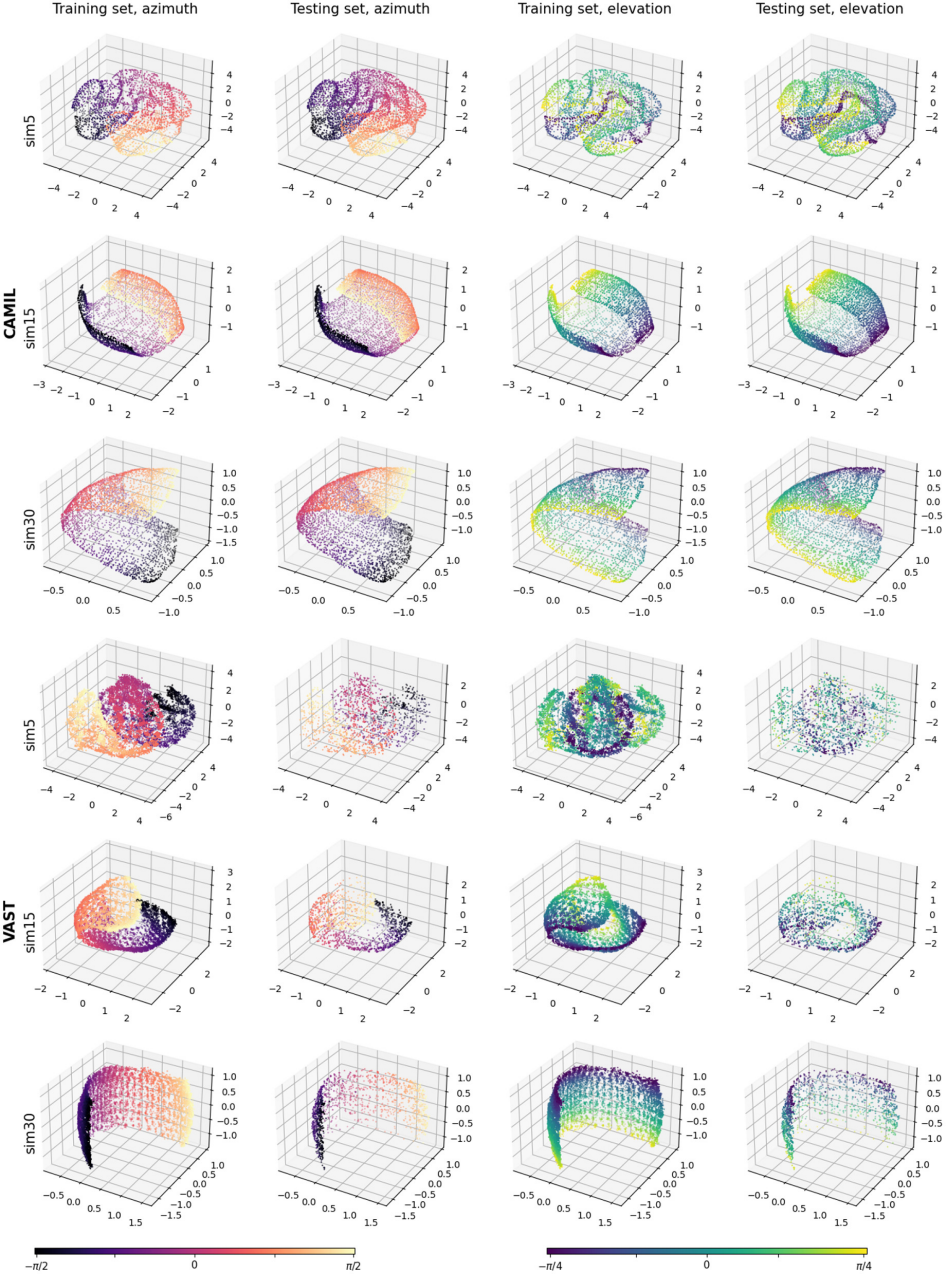
Visualizations of the WSCE embeddings. Column 1 and 2 are azimuth training and testing embeddings. Column 3 and 4 are elevation training and testing embeddings.

To explore the learned latent space structure, we test several similarity threshold angles ϵ_*u*_∈{5°, 15°, 30°}, indicated as “_sim5,” “_sim15,” and “_sim30,” respectively. Since when calculating the similarity labels, we first normalize the relative source location coordinates to have unit norm (i.e., source coordinates are relocated to have unit distance to the receiver), chosen the similarity threshold angles yield the following similarity threshold for the physical source distance: ϵ_*s*_∈ {0.09, 0.26, 0.52 m}. Figure 5 shows the training set embeddings and the testing set embeddings for the CAMIL testing set and the VAST testing-set-1. Firstly, it can be observed that the proposed WSCE method learns a manifold from the binaural cues that can reflect the sound source location without any azimuth/elevation annotations. This manifold has a clear structure and a similar structure is obtained in both the CAMIL dataset (with reverberant speech) and the VAST dataset (with varying reverberation). Secondly, when using smaller similarity threshold angles (i.e., ϵ_*u*_ = 5°), the structure of the manifold tends to become irregular and folded, and when using larger threshold angles (i.e., ϵ_*u*_ = 15° and ϵ_*u*_ = 30°), the structure of the manifold tends to become smooth and unfolded. Elaborating the intuition introduced in Section 4.3, this may be due to the fact that when the similarity threshold angle is small, the contrastive loss has a small range of action on penalizing mislocated dissimilar pairs, resulting in many dissimilar pairs not being subject to repulsive forces, and instead, similar pairs are attracted and clustered in local areas. When a large similarity threshold angle is used, each embedding is subject to both attractive and repulsive forces from a large number of other embeddings, thus maintaining an overall uniformly equilibrium state in the global perspective.

**Figure 6:**
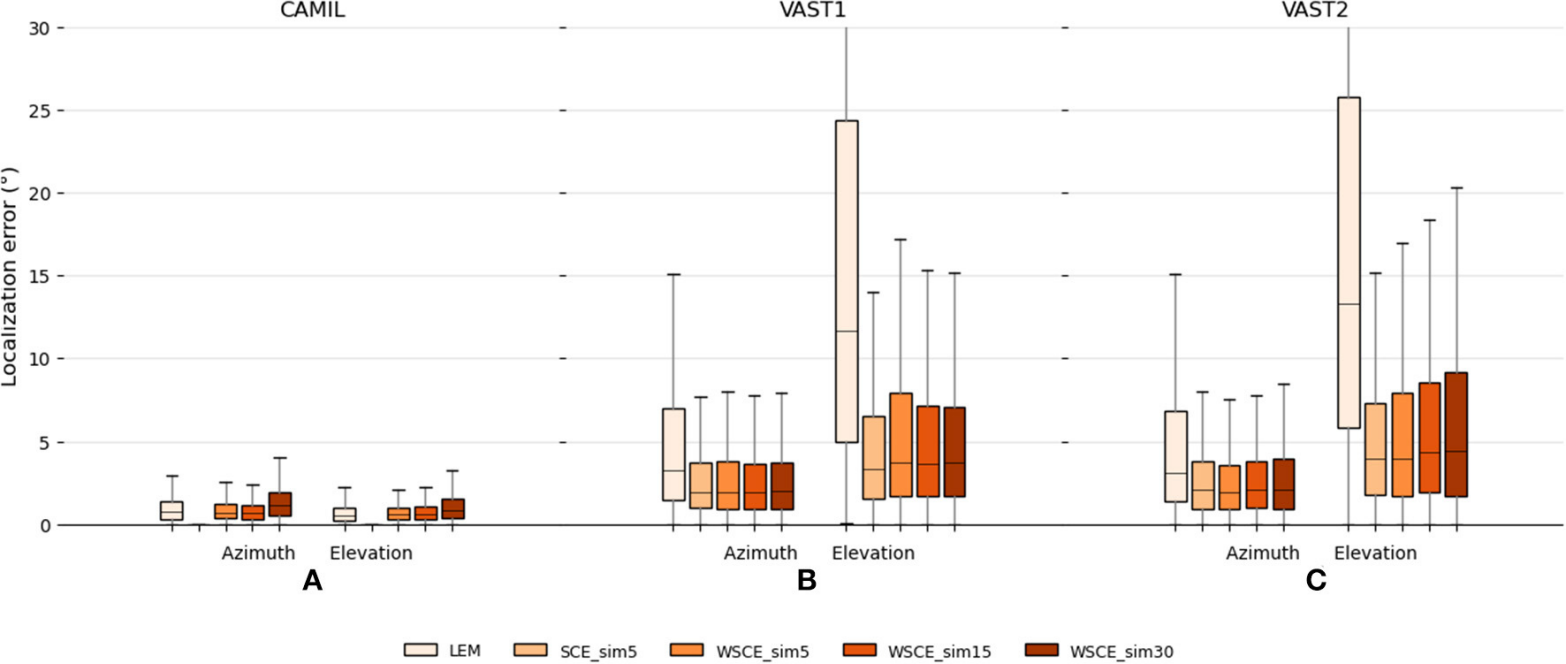
Localization performance on the CAMIL testing set **(A)**, the VAST testing-set-1 **(B)**, and the VAST testing-set-2 **(C)** for the LEM, the SCE and the WSCE methods.

In addition to the above mentioned qualitative experiments, we also conduct quantitative experiments to use the WSCE for source localization and compare the results to the LEM embeddings and the SCE_sim5. The localization results are shown in Figure 6. In the fixed acoustic condition with the CAMIL dataset, the SCE_sim5 still performs the best but it trains separate embeddings for azimuth and elevation. In contrast, both the proposed WSCE and the LEM embedding train one embedding for both azimuth and elevation estimation and show a strong source localization ability as well. In azimuth estimation, the WSCE_sim15 performs slightly better than the WSCE_sim5, then followed by LEM and WSCE_sim30 (achieving median errors equal to 0.64, 0.69, 0.72, and 1.16°, respectively). In elevation estimation, LEM exhibits a median error equal to 0.49° and performs slightly better than the WSCE_sim15 and WSCE_sim5, which have the same median error equal to 0.58°. WSCE_sim30 performs worst in elevation estimation and achieves a median error equal to 0.82°. Nevertheless, the WSCE shows a comparable localization ability to the LEM in the fixed acoustic condition.

In varying acoustic conditions with the VAST dataset, instead, the WSCE shows a much lower localization error than the LEM embeddings and it is even approaching the SCE_sim5 performance. Firstly, with the VAST testing-set-1, the WSCE_sim5, WSCE_sim15, and WSCE_sim30 perform equally well (azimuth median errors equal to 1.96, 1.94, and 1.98°, respectively, and elevation median errors equal to 3.69, 3.64, and 3.69°, respectively), and the SCE_sim5 has slightly better elevation estimation than either WSCE method (achieving azimuth and elevation median errors equal to 1.96 and 3.32°, respectively). For the VAST testing-set-2, similarly, the WSCE_sim5, WSCE_sim15, WSCE_sim30, and SCE_sim5 perform somewhat equally well (achieving azimuth median errors equal to 1.93, 2.1, 2.1, and 2.1°, respectively, and elevation median errors equal to 3.99, 4.34, 4.44, and 3.94°, respectively). Although the unidimensional SCE_sim5 and the WSCE with a small similarity threshold show narrower interquartile range than other methods, we do suggest to use a similarity threshold angle ϵ_*u*_ = 15 ° for WSCE to achieve both good visualization and localization performance.

Secondly, the WSCE largely outperforms the LEM embeddings in varying acoustic conditions where LEM only obtains an azimuth median error of 3.28° and an elevation median error of 11.7° for VAST testing-set-1, and an azimuth median error of 3.09° and an elevation median error of 13.27° for VAST testing-set-2, respectively. Also, the WSCE has a much narrower interquartile range than the LEM, which may indicate that the proposed WSCE is more robust to reverberation than the LEM embeddings.

### 5.5. WSCE with unseen HRTFs

To further verify the generalization capability of the proposed WSCE, we test the WSCE with different HRTFs that are not seen during the training. To create simulated binaural recordings, we use the CIPIC dataset (Algazi et al., 2001), which consists of 45 real-life measured HRTFs. There are in total 45 subjects (43 human subjects and 2 dummy head subjects), and for each subject, 1250 HRTFs are measured for each ear and from different azimuth and elevation angles. We select azimuth and elevation angles in range the [−90, 90°] and [−45, 45°], respectively, corresponding to the other datasets mentioned in the former sections. The HRTFs are then convoluted with simulated reverberant recordings (excited by 2 s white noise). Those recordings are generated using the image method (Allen and Berkley, 1979), in a shoebox room that has dimension 3.5 × 5 × 2.8 m, and reverberation time equal to 0.3 s.

**Figure 7:**
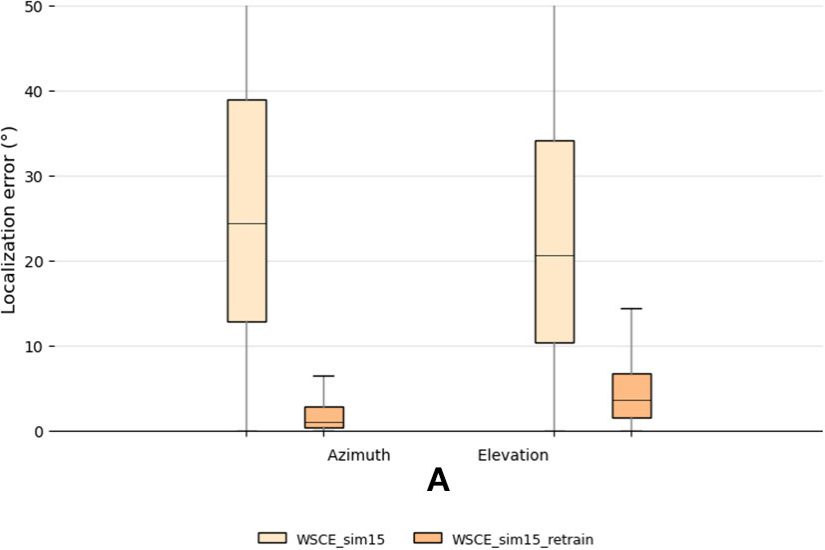
Testing set localization performance for the WSCE_sim15 and WSCE_sim15 retrained with 33 different HRTFs other than the once used in generating test recordings.

We randomly select recordings from 10 subjects for testing, and use WSCE_sim15 for estimating their source locations. The localization results are plotted in Figure 7. Since we train the WSCE_sim15 only using one HRTF, the model could not generalize well to recordings made with unseen HRTFs. Therefore, we observe a dramatic performance degradation, in which the median errors of azimuth and elevation localization are 24.3 and 20.1°, respectively.

To overcome the performance degradation, we propose two approaches:

1. Personalized training (user-dependent): this approach is especially interesting for hearing-aid applications since the hearing-aid is designed for a specific user, and it is not shared with different people. Therefore, the HRTF of the designated user can be measured and be used in the model training or fine-tuning process to create a user-dependent model.
2. Increase training data variety (user-independent): another solution consists in using more HRTFs to create the training data for training the WSCE. Then, the trained model can generalize to people with different HRTF than the ones in training data. A rule of thumb is that the higher the variety of the training data (with annotation), the better the generalization capability of the model.

We adopt the second approach to retrain the WSCE_sim15 and use the rest of the HRTFs from the CIPIC dataset, which are different from the data used in the testing (i.e., user-independent). This results in 33 HRTFs that are used for training, 2 for validation and 10 for testing. We also simulate random shoebox rooms that have reverberation time between 0.1 and 0.4 s. The localization error of the retrained model is shown in Figure 7 with name “WSCE_sim15_retrain.” The azimuth and elevation median errors of the retrained model have been largely reduced from 24.3 to 1.1° and 20.1 to 3.6°, respectively, showing the effectiveness of this approach.

**Figure 8:**
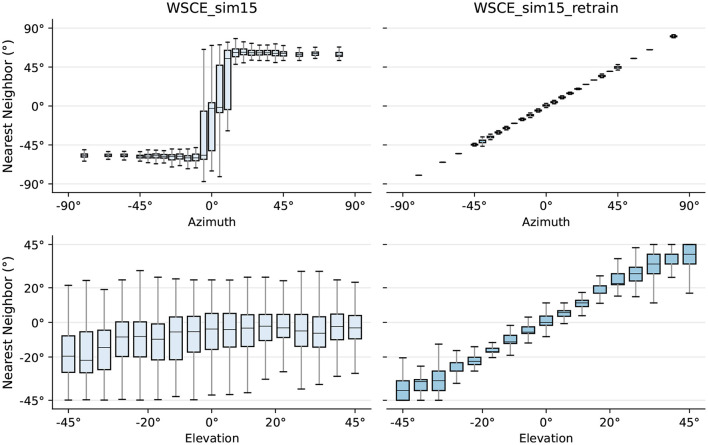
True locations of sources in the testing set versus the locations of their respective nearest neighbor in the training set for WSCE_sim15 and WSCE_sim15 retrained with 33 different HRTFs other than the once used in generating test recordings (i.e., WSCE_sim15_retrain).

We further analyse the relationship between the CIPIC testing set embeddings and their respective nearest training set neighbors and illustrate the results in Figure 8. The X-axis is the true azimuth or elevation angle of the testing embedding, and the Y-axis is the location of the corresponding nearest training set neighbor predicted by the WSCE_sim15 which is summarized using box plots. For the original WSCE_sim15, the median nearest neighbor location angles are shifted compared to the true testing location in the case of both azimuth and elevation angles, and the interquartile ranges of the nearest neighbor location angles are large, indicating that the neighbors of the original WSCE_sim15 are poorly preserved, which also suggests that the original WSCE_sim15 trained with only one HRTF cannot be generalized to the unseen HRTFs. In contrast, WSCE_sim15_retrain preserves the neighborhoods much better because the median of the location angles of the nearest neighbors predicted by the WSCE_sim15_retrain is close to the true location angle of the test embedding. In addition, compared to the original WSCE_sim15 model, the location angle of the nearest neighbor predicted by the WSCE_sim15_retrain has a smaller interquartile range. In summary, the generalization to unseen HRTFs is much better after retraining WSCE_sim15 with 33 real-life HRTFs.

However, a limitation of our simulations is that we use synthetic rooms with slightly different acoustic properties than real-life rooms. In addition, we always excite the sound source with white noise, which has a broadband spectrum, while real-life sounds may not have the same characteristics. We propose to increase the variety of training data covering real-life conditions, using more HRTFs recorded at finer azimuth/elevation angles, and using Room Impulse Responses (RIRs) from more complex rooms, which we believe will further improve the generalization capability of the proposed WSCE model.

## 6. Conclusions

We proposed a DNN framework for supervised dimensionality reduction of binaural cue measurements, followed by a nearest-neighbor regression method for source localization. Our manifold-learning-based method has better binaural sound source localization performance than the baseline manifold learning method in both know and unknown reverberant conditions and in a small training set condition. In comparison with a feed-forward learning method, our proposed method not only provides a better visualization ability, but also achieves a similar or better performance in binaural sound source localization. Moreover, our proposed method can capture a smooth manifold structure for low data density regions and outperforms the baseline manifold learning method and the feed-forward method in case of a small amount of training data.

In addition to the supervised dimensionality reduction method, we also proposed a weakly supervised embedding, i.e., WSCE, that only requires implicit latent space proximity labels for training. This WSCE can simultaneously estimate the azimuth and elevation of the sound source, and is also robust to unknown reverberation. Quantitative experimental results demonstrate that this WSCE has almost similar localization performance as the supervised method, and it performs much better than the traditional unsupervised embedding in varying acoustic conditions.

To further increase the generalization capability of the proposed model, we hope to learn the SCE and WSCE embeddings with big variety of training data covering more real-life conditions, such as using more HRTFs recorded at finer azimuth/elevation angles and using RIRs from more complex rooms. In addition, we also aim to further investigate how to apply the proposed SCE and WSCE in data synthesis. When combining these methods with a generative model, we speculate that the embeddings can be used to synthesize binaural features or even audio waveforms to aid data-driven binaural source localization models.

Since potentially applicable systems for the proposed model (e.g., hearing aids) often have limited computational resources, reducing the model complexity and the number of model parameters is therefore a relevant direction for future research. Possible approaches to achieve this include model pruning (i.e., removing the DNN neurons that are associated with very small weights), model information distillation (Hinton et al., 2015) and model parameter quantization.

## Data availability statement

The datasets generated and/or analysed during the current study are available in the CAMIL repository (https://team.inria.fr/perception/the-camil-dataset), and the VAST repository (http://thevastproject.inria.fr/dataset).

## Author contributions

DT and MT invented the concept of estimating binaural sound source location in reverberant acoustic conditions using the *siamese* neural network with a contrastive loss, designed and interpreted the computer simulations. DT, MT, and TW jointly developed the research methodology to turn this concept into a usable and effective algorithm. DT implemented the computer simulations and MT managed to the dataset used. All authors contributed in writing the manuscript, and further read and approved the final manuscript.

## Funding

DT was sponsored by the Chinese Scholarship Council (CSC) (no. 201707650021). MT was a Postdoctoral Fellow of the Research Foundation Flanders—FWO-Vlaanderen (no. 12X6719N). This research work was carried out at the ESAT Laboratory of KU Leuven. The research leading to these results has received funding from the KU Leuven Internal Funds C2-16-00449 and VES/19/004, and the European Research Council under the European Union's Horizon 2020 research and innovation program/ERC Consolidator Grant: SONORA (no. 773268). This paper reflects only the authors' views and the Union is not liable for any use that may be made of the contained information.

## Conflict of interest

The authors declare that the research was conducted in the absence of any commercial or financial relationships that could be construed as a potential conflict of interest.

## Publisher's note

All claims expressed in this article are solely those of the authors and do not necessarily represent those of their affiliated organizations, or those of the publisher, the editors and the reviewers. Any product that may be evaluated in this article, or claim that may be made by its manufacturer, is not guaranteed or endorsed by the publisher.
